# Using ArcMap, Google Earth, and Global Positioning Systems to select and locate random households in rural Haiti

**DOI:** 10.1186/1476-072X-12-3

**Published:** 2013-01-18

**Authors:** Peter J Wampler, Richard R Rediske, Azizur R Molla

**Affiliations:** 1 Department of Geology, Grand Valley State University, 1 Campus Dr, Allendale, MI 49401, USA; 2 Annis Water Resource Institute, Grand Valley State University, 740 W. Shoreline Drive, Muskegon, MI, 49451, USA; 3 Department of Anthropology, Grand Valley State University, 1 Campus Drive, Allendale, MI, 49401, USA

**Keywords:** Bacterial water testing, Google Earth, Ethnographic study, Anthropology, GPS

## Abstract

**Background:**

A remote sensing technique was developed which combines a Geographic Information System (GIS); Google Earth, and Microsoft Excel to identify home locations for a random sample of households in rural Haiti. The method was used to select homes for ethnographic and water quality research in a region of rural Haiti located within 9 km of a local hospital and source of health education in Deschapelles, Haiti. The technique does not require access to governmental records or ground based surveys to collect household location data and can be performed in a rapid, cost-effective manner.

**Methods:**

The random selection of households and the location of these households during field surveys were accomplished using GIS, Google Earth, Microsoft Excel, and handheld Garmin GPSmap 76CSx GPS units. Homes were identified and mapped in Google Earth, exported to ArcMap 10.0, and a random list of homes was generated using Microsoft Excel which was then loaded onto handheld GPS units for field location. The development and use of a remote sensing method was essential to the selection and location of random households.

**Results:**

A total of 537 homes initially were mapped and a randomized subset of 96 was identified as potential survey locations. Over 96% of the homes mapped using Google Earth imagery were correctly identified as occupied dwellings. Only 3.6% of the occupants of mapped homes visited declined to be interviewed. 16.4% of the homes visited were not occupied at the time of the visit due to work away from the home or market days. A total of 55 households were located using this method during the 10 days of fieldwork in May and June of 2012.

**Conclusions:**

The method used to generate and field locate random homes for surveys and water sampling was an effective means of selecting random households in a rural environment lacking geolocation infrastructure. The success rate for locating households using a handheld GPS was excellent and only rarely was local knowledge required to identify and locate households. This method provides an important technique that can be applied to other developing countries where a randomized study design is needed but infrastructure is lacking to implement more traditional participant selection methods.

## Background

Use of a geographic information system (GIS), a system for input, storage, manipulation, and output of geographic information provides a powerful tool for public health assessment and monitoring in remote locations and developing countries [1]. GIS-based approaches have been used to study infectious diseases like malaria in Africa [2] and dengue virus in different parts of the world [3,4]. Effective use of GIS in public health assessment typically requires basic infrastructure data layers for georeferencing data (addresses, zip codes, streets, city blocks, location of health facilities, etc.). In rural Haiti, geospatial infrastructure often is lacking, making it difficult to implement GIS-based household water quality sampling, ethnographic surveys, and randomized study designs. Although satellite imagery and aerial photos have been available for many years, their use was limited due to cost and availability, particularly for public health officials in developing countries [5,6]. The advent of internet based mapping technologies such as Google Earth^TM^ and Google Maps^TM^ provide free satellite imagery, aerial photos, and topographic data for most of Earth's land surface, resulting in increased availability of mapping technology for use by public health workers and researchers [7-9]. However, Google Earth^TM^ still lacks the robust map manipulation and analysis functions of GIS software [9], and often requires data export/import to a GIS program such as ESRI’s ArcMAP 10.0 (ESRI, Redlands, CA) for further analysis and map preparation. Recently, public health assessment programs have effectively combined GIS and Google Earth^TM^ to collect data for dengue fever [10], schistosomiasis [11], and mortality [12]. This article describes a method that utilizes GIS; Google Earth^TM^ (Google Inc., Mountain View, CA), and Microsoft Microsoft Excel^TM^ 2010 (Microsoft Corp., Redmond, WA) to map and select random households for ethnographic surveys and water sampling in rural Haiti. The technique is applicable to other developing countries, does not require access to address records or ground-based surveys to collect household location data, can be prepared prior to field work with common software packages, and requires only a handheld GPS to accomplish accurate field location of selected households.

Random sampling for ethnographic and public health surveys provides a representative sample of target populations, and ultimately representative data from these populations can be collected [13]. In cases where the sample population is inherently stratified or grouped, stratified random sampling can be employed [14]. For example, populations can be stratified by age, gender, or geographic location. Random samples, subjects, or households, are then selected from each strata or group. Stratification for this study was geographically-based. Clusters or groups of homes were located within circular polygon regions (strata) variable distances (1–9 km) from Hôpital Albert Schweitzer (HAS) [15], a local source of health care and community development support. Homes were randomly selected from each cluster for ethnographic surveys and water sampling.

### Study area

**Figure 1:**
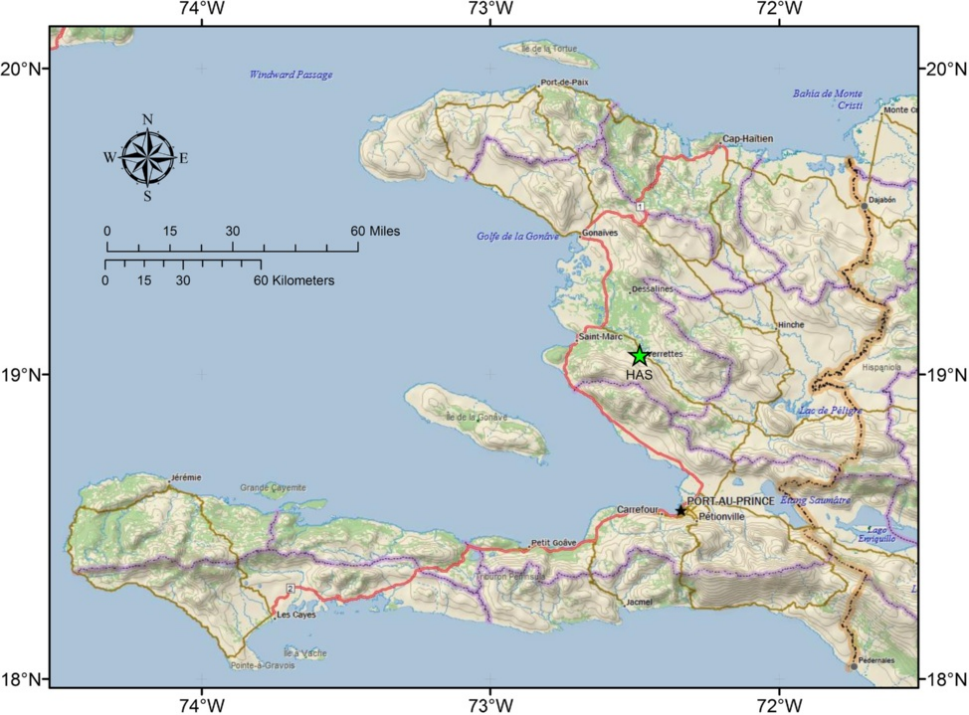
Study area location map.

The study site was located near HAS in Deschapelles, Haiti, roughly 65 kilometers north northwest of Port au Prince, Haiti (see Figure 1). Haiti is a mountainous country of rough terrain, sparse road infrastructure, and few major rivers. The Artibonite River, the largest river in Haiti, flows below the study area and is served by a paved highway which connects Port au Prince with the Central Plateau. The Artibonite River valley was the location of the initial cholera outbreak in 2010 [16]. There are no paved roads in the mountains above Deschapelles and streets and roads are often not named or labeled. Many roads become impassable by vehicles after precipitation events due to river crossings, steep terrain, and lack of traction on slick limestone surfaces. Homes in rural Haiti are typically constructed of rock and wood, and are arranged in loose aggregates or villages near water sources, roads, or along ridge tops. Many of the homes are only accessible by foot path. Community and village names often have multiple spellings and pronunciations depending on the context and source. Village households sometimes have addresses written on door posts; however addresses are not related to a regular grid or street and may not be recorded or documented in a form that is accessible for participant selection and implementation of a random survey.

## Results and discussion

The list of homes and mapping were used to create laminated field sheets to be used in concert with handheld GPS units to locate homes for interviews and water sampling. A total of 55 homes were visited during the 10 days of fieldwork. Over 96% of the homes mapped using Google Earth imagery were correctly identified as occupied dwellings (see Table 1). Only 3.6% of the occupants of mapped homes visited declined to be interviewed. 16.4% of the homes selected were not occupied at the time of the visit. Occupancy was especially problematic on market days when many residents travel to nearby cities to sell and buy goods. The handheld GPS units proved very effective at field location of homes with virtually 100% successful location of pre-selected structures.

**Table 1:** Home clusters and summary data for field data collection from 55 homes

**Distance to HAS**	**Geographic name**	**Homes in Cluster**	**Homes sampled**	**% sampled**	**Homes misidentified**	**Homes declined**	**Not home**
1	LaForge	69	5	7%	0	1	0
2	Haute LaForge/Ange	44	7	16%	0	0	0
3	Ange	44	7	16%	0	0	2
4	Champion/Vielot	71	7	10%	2	1	0
5	Savonet	63	7	11%	0	0	2
6	Salo	40	6	15%	0	0	1
7	Salas	48	6	13%	0	0	2
8	Trankite	38	6	16%	0	0	0
9	Dauphine	53	4	8%	0	0	2

Factors that contributed to unsuccessful home identification, home location, and occupancy at the time of our visit included: 1) lack of homeowner availability on market days and holidays; 2) house construction methods in Haiti; and 3) road and trail access. Market days in much of Haiti take place two days a week in a given village and often occur on different days in other villages within walking distance. This results in an exodus of residents from their homes in certain rural villages on market days. Since some of these villages are remote and difficult to access, it is prudent to coordinate visitation of these locations to avoid market days and holidays. It is common practice in Haiti for homes to be “under construction” for years to decades. This may result in a roof being put in place for many years prior to occupancy of a dwelling. Aerial mapping of homes was unable to distinguish between roofs of homes under construction and occupied dwellings, resulting in at least two cases in the mapping of homes which were not occupied. In some cases, dwellings that appeared to be close together were in reality difficult to access due to a lack of roads or trails which were not evident from aerial mapping.

Due to the variable nature of the size and configuration of houses, schools, and churches in Haiti, it was not feasible to distinguish them from individual homes using aerial imagery. Consequently it was important to identify a larger number of homes from which the random sample could be selected. Our drivers, interpreter, and local residents were able to provide valuable local knowledge in these cases to locate expedient access routes and facilitate sampling in a timely fashion.

## Conclusions

The method used to generate and field locate random homes for surveys and water sampling was an effective means of selecting random households in a rural environment lacking geolocation infrastructure. The success rate for locating households using a handheld GPS was excellent and only occasionally was local knowledge required to identify and locate households. The use of this method avoided the potential bias which could be introduced if an interpreter or driver were used to locate homes for survey or sampling. This method was fast, relatively straightforward and required only limited access to GIS and computer technology to setup the field survey. Once the lists and GPS coordinates were generated and loaded to field units, no internet or cell phone data access was needed to accomplish the survey. This method provides an important technique that can be applied to other developing countries where a randomized study design is needed but the necessary geolocation infrastructure is lacking to implement a more traditional randomized study design.

## Methods

Home clusters were chosen based on distance from HAS. One of the goals of the ethnographic survey was to evaluate how proximity to this important source of health care, community development services, and water quality interventions influenced water quality perceptions and practices. Cluster selection was restricted to two geographic areas, similar in size to counties in the United States, called Belanger and Bastien. Cluster locations were all within 9 kilometers of HAS and were chosen based on 1) household density; 2) road access; and 3) field data collection feasibility. Clusters had to be located so that water quality bacterial analysis could be completed within the required holding time of 6 hours to maintain *E. coli* viability [17]. Occasionally sites were revisited on the way to or from another location. This was made possible by having the sites located in the same direction from the hospital.

We received Grand Valley State University Institutional Review Board (IRB) approval for the water sampling and ethnographic surveys (IRB# 10-256-H) associated with this study. The random selection of households and the location of these households during field surveys were accomplished using GIS, Google Earth^TM^, Microsoft Excel^TM^, and handheld Garmin GPSmap 76CSx GPS units. Since there were little or no written documents available from the Haitian government, with respect to census data and residential addresses, the development of a remote selection method was essential to the success of the study. The method could be successfully applied in other developing countries where geographic infrastructure is lacking.

### ArcMAP buffers and household mapping

**Figure 2:**
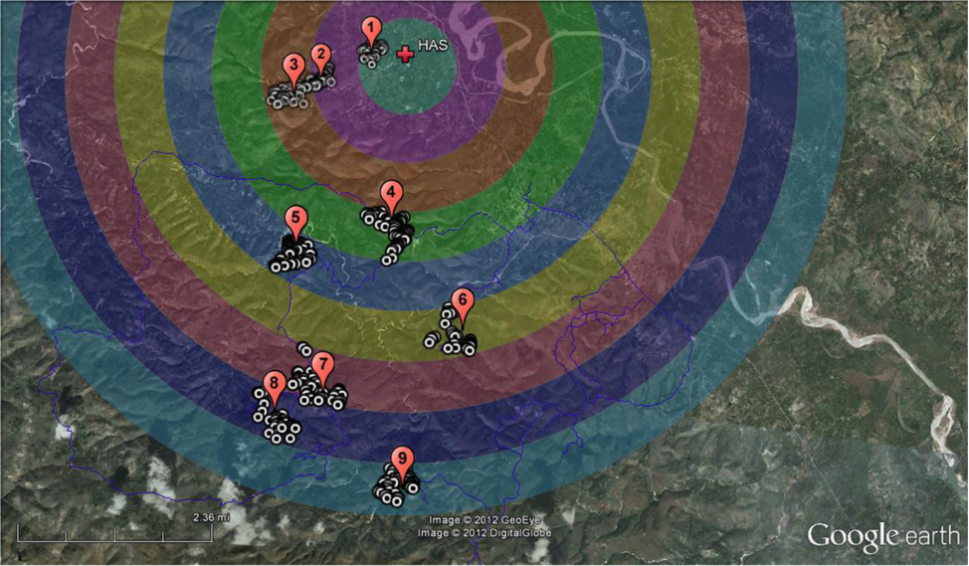
**One kilometer circular buffer regions centered on the Hôpital Albert Schweitzer (HAS). **Basemap layer is a Landsat Enhanced Thematic Mapper true color image (bands 1,2,3).

A Multi-band Landsat Enhanced Thematic Mapper (ETM+) satellite image, captured August 21, 2000 and obtained from the EarthExplorer web site, was used as a basemap to create geographically stratified circular regions [18]. The radius of each circular region was increased by 1 kilometer from HAS using the ArcMAP 10 BUFFER command to create a set of nested circular regions (Figure 2). The circular polygon regions were then exported as a KML file into Google Earth™. This allowed for the use of recent high resolution Google Earth™ imagery to identify clusters of households and map individual dwellings within each cluster for later random selection and sampling. Google Earth aerial photos were taken in late 2010, had excellent resolution (~1-3 m), and was sufficiently detailed to map individual homes with very good accuracy.

**Figure 3:**
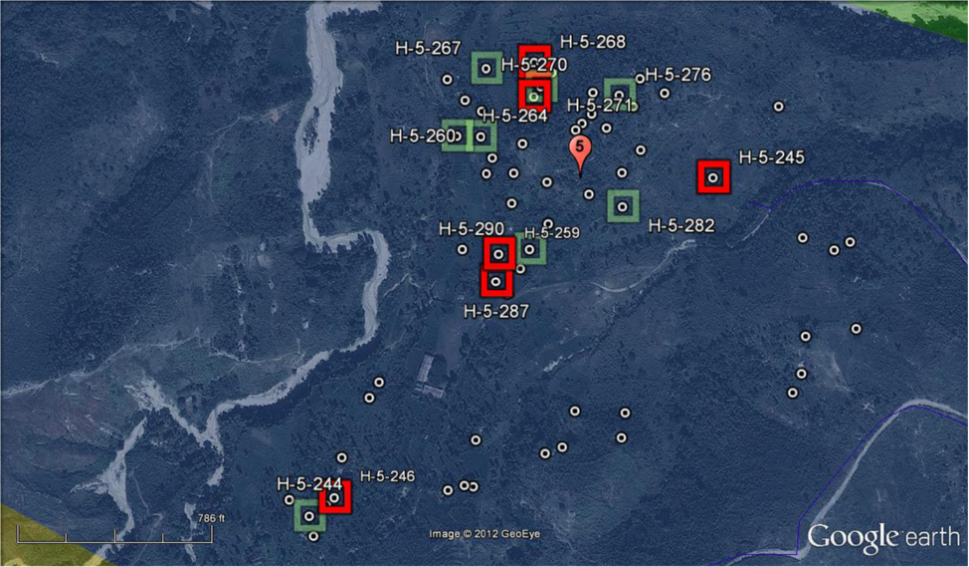
**Example of a Google Earth aerial photo onto which homes were mapped (white dots). **Green Boxes are the homes randomly selected using Microsoft Excel and the red boxes are households actually visited during the survey.

In order to facilitate timely field sampling and surveys, clusters of homes within each of nine circular buffer regions were selected and individual homes were mapped using pushpins in Google Earth (total of 537 houses) (see Figure 3). Areas of high home density were chosen to facilitate timely interviews and access within the limited time available for surveys and subsequent water analysis. Approximately 50 homes were mapped in each cluster from which a subset was chosen randomly for the ethnographic surveys.

The point layer of homes created in Google Earth was imported into ArcMap and attributes were added for latitude and longitude (WGS 84 coordinate system). These geographic coordinates were later uploaded into a handheld GPS and used to locate homes in the field for conducting field surveys.

### Random household selection and field location

The entire attribute table from ArcMap was exported to Microsoft Excel and a column was added for a unique identification number (for example H-1-1) which would be used for coding ethnographic surveys and water samples (see Table 2). A column also was added so that each household could be assigned a random number between 0 and 1 using the RAND() function in Microsoft Excel. The sorting function then was used to perform a multi-level sort of all the households first by cluster then by random number. The end result was a list of mapped households in random order for each cluster. For the purposes of the study, the first twelve households randomly selected from each cluster were designated for surveys and water analysis. The entire process of home identification, mapping, field map production, and random list generation took approximately 8–10 hours. WGS84 latitude and longitude coordinates were uploaded to a Garmin GPSmap 76CSx. Each of the first 12 random homes was located using the GPS until 5–9 surveys were completed in a given cluster. If no one was home at a selected household, or the homeowners were unavailable, the next home on the random list was selected.

**Table 2:** Household spreadsheet for area 1 (one kilometer from HAS)

**OBJECTID**	**GVSU_ID**	**Sample_Order**	**Random_no**	**Longitude**	**Latitude**
1	H-1-31	1	0.01149229844	-72.498970817	19.076093979
2	H-1-34	2	0.01827961793	-72.499270420	19.076230928
3	H-1-18	3	0.05403995752	-72.499427800	19.074163608
4	H-1-25	4	0.05975003586	-72.497521680	19.075499980
5	H-1-43	5	0.08377157472	-72.496706672	19.076214581
6	H-1-32	6	0.08571745333	-72.498367128	19.075961350
7	H-1-37	7	0.08664302715	-72.499569703	19.075585606
8	H-1-56	8	0.12194280027	-72.498046910	19.076895770
9	H-1-39	9	0.14165299621	-72.499963138	19.075850242
10	H-1-52	10	0.19335796388	-72.498023657	19.076108015
11	H-1-33	11	0.19348332138	-72.499451467	19.076067500
12	H-1-17	12	0.19705891490	-72.499290284	19.074004758
13	H-1-6	13	0.19907687246	-72.498054155	19.074578809
14	H-1-46	14	0.21060435716	-72.496620005	19.076746775
15	H-1-7	15	0.25007727144	-72.497646039	19.073284607
16	H-1-44	16	0.26452753465	-72.497272413	19.076373471
17	H-1-45	17	0.27173287408	-72.497747780	19.076187511
18	H-1-13	18	0.27507139853	-72.497780219	19.074519407
19	H-1-26	19	0.28955065050	-72.497639192	19.075524139
20	H-1-62	20	0.29334220958	-72.496385904	19.077126559
21	H-1-5	21	0.29419897483	-72.497958235	19.074562629
22	H-1-66	22	0.29790014023	-72.495913123	19.076722700

This table has already been sorted by the random number column to produce a random order for Household ID’s.

## Competing interests

The authors declare that they have no competing or financial interests in any of the technologies described in this paper. No other manuscript contains the same, similar, or related information, and this manuscript is not being considered for publication elsewhere. We appreciate your consideration of our article and please contact us if you have questions.

## Authors’ contributions

PW performed all the Google Earth and ArcMap data manipulation and map preparation. AM participated in GPS field location of homes and ethnographic surveys; and RR collected and analyzed water samples. All three authors contributed to the manuscript preparation and review. All listed authors made substantive intellectual contributions to the methods, fully participated in the study, and accept full responsibility for the work. All authors read and approved the final manuscript.
